# Association between Dietary Magnesium Intake and Radiographic Knee Osteoarthritis

**DOI:** 10.1371/journal.pone.0127666

**Published:** 2015-05-26

**Authors:** Chao Zeng, Hui Li, Jie Wei, Tuo Yang, Zhen-han Deng, Ye Yang, Yi Zhang, Tu-bao Yang, Guang-hua Lei

**Affiliations:** 1 Department of Orthopaedics, Xiangya Hospital, Central South University, Changsha, Hunan Province, China; 2 Department of Epidemiology and Health Statistics, School of Public Health, Central South University, Changsha, Hunan Province, China; University of Padova, ITALY

## Abstract

**Objective:**

To examine the cross-sectional associations between dietary magnesium (Mg) intake and radiographic knee osteoarthritis (OA), joint space narrowing (JSN), and osteophytes (OST) respectively.

**Methods:**

A total of 1626 subjects were included in the study. Dietary intake was assessed using a validated semi-quantitative food frequency questionnaire. Radiographic knee OA was defined as Kellgren-Lawrence (K-L) Grade 2 in at least one leg. JSN and OST were assessed individually according to the Osteoarthritis Research Society International (OARSI) atlas. A multivariable logistic analysis model was applied to test the various associations after adjusting for potentially confounding factors.

**Results:**

The relative odds of radiographic knee OA were decreased by 0.53 times in the third quintile of Mg intake [odds ratio (OR) 0.53, 95% confidence interval (CI) 0.28–1.01], 0.40 times in the fourth quintile (OR 0.40, 95% CI 0.17–0.94) and 0.34 times in the fifth quintile (OR 0.34, 95% CI 0.11–1.00) compared with those in the lowest quintile, while *P* for trend was 0.111. The relative odds of JSN were decreased by 0.49 times in the third quintile of Mg intake (OR 0.49, 95% CI 0.28–0.88) and 0.37 times in the fifth quintile (OR 0.37, 95% CI 0.14–0.98) compared with those in the lowest quintile, while *P* for trend was 0.088. There was no significant relationship between dietary Mg intake and the presence of OST.

**Conclusions:**

The findings of this cross-sectional study indicate that Mg intake is inversely associated with radiographic knee OA and JSN. It supports potential role of Mg in the prevention of knee OA.

**Level of Evidence:**

LevelIII, cross-sectional study.

## Introduction

Osteoarthritis (OA) is a progressive rheumatic disease whose incidence is growing continuously with the aging populations in many societies. In Asia, the prevalence of knee OA ranges from 11.8% to 55.5% in those ≥ 40 years of age [1–6]. In Japan, it was estimated that 25,300,000 people are affected by radiographic knee OA [4]. Along with the further increase in the prevalence of knee OA [7], there is an increasing rate of knee arthroplasty performed, which is currently the only effective treatment for the late phase of OA. This fact has made identification of effective conservative treatments and preventive methods a high priority. Effective therapies and prevention options for knee OA, however, are limited. Among the factors potentially useful for preventing knee OA is magnesium (Mg) intake. Mg is an essential micronutrient for humans.

Low-grade systemic inflammation may play an important role in the pathophysiology of OA [8,9]. Previous studies revealed that low dietary Mg intake was associated with elevated serum C-reactive protein (CRP) [10–14], which is the most sensitive biomarker for low-grade systemic inflammation. Animal studies also indicated that some proinflammatory cytokines (interleukin-6, tumor necrosis factor α) were increased under Mg deprivation [15]. There is also a strong correlation between Mg and the immune response [16]. Activation of cells (e.g., macrophages, neutrophils, endothelial cells) was reported to be associated with Mg deficiency as well [17,18]. Low Mg intake may be a contributing factor to the development of OA through inflammatory and/or immune mechanism.

In 2003, Hunter et al. [19] observed a significant decrease in serum Mg in the OA patient of the twins. Low serum Mg levels were also detected in women residing in OA-endemic areas [20]. Recently, Qin et al. [21] reported that there was a modest inverse threshold association between Mg intake and radiographic knee OA in Caucasians but not in African Americans. It has also been found that OA was related to diabetes [22,23] and hypertension [22,24–27], but the multivariable logistic regression model devised by Qin et al. [21] was not adjusted for diabetes or hypertension. Differences may also exist among different populations. For example, several epidemiological studies suggested a significant inverse association between dietary Mg intake and the risk of developing diabetes in western populations [28–31] but not in Japanese people [32]. The Kellgren-Lawrence (K-L) radiographic atlas is based on the radiographic presence of cartilage destruction and osteophytes (OST), which are different, unrelated abnormalities. In view of the above-described literature research, the present cross-sectional study aimed to examine (1) whether there is an association between dietary Mg intake and radiographic knee OA in the Chinese population; and (2) whether dietary Mg intake is associated with joint space narrowing (JSN) and/or the presence of OST in the same population.

## Materials and Methods

### Study population

We obtained approval for this study from the ethics committee at Xiangya and third-Xiangya Hospital, Central South University. Also, we obtained written informed consent from the patients in our study. The Xiangya Hospital Health Management Center Study (XYHMCS) included a cohort consisting mainly of apparently healthy Chinese people from general public for health screening. This overall XYHMCS mainly aimed to explore the risk factors (e.g., dietary factors, serum micronutrients level, lifestyle behaviors) of various diseases, such as OA, hyperuricemia, and so on. The study design has been published previously [33]. Routine health checkups are very common in China, because the Chinese government encourage people to take periodic medical examinations. Registered nurses interviewed all participants during the examination using a standard questionnaire, with the purpose to collect information on demographic characteristics and health-related habits. Subjects were selected according to the following inclusion criteria: 1) 40 years old or above; 2) undergoing weight-bearing bilateral anteroposterior radiography of the knee; 3) completion of the semi-quantitative food frequency questionnaire (FFQ) about the average consumption of foods and drinks over the past 1 year; 4) availability of all basic characteristics, including age, gender, body mass index (BMI), smoking status, etc. In the beginning, this cross-sectional study included 2364 subjects who were undergoing routine checkups including weight-bearing bilateral anteroposterior radiography of the knee at the Department of Health Examination Center Xiangya Hospital, Central South University in Changsha, Hunan Province, China, from October 2013 to January 2014. Then, individuals with other joint diseases with radiographic evidence, such as osteochondroma or fracture (n = 10), or with missing data of certain characteristics or physical examinations, such as BMI and blood pressure (n = 139), or with missing data of biochemical tests, such as blood glucose (n = 57), or younger than 40 years old (n = 211), were excluded. The overall response rate of the survey was 83.5%, with a total of 321 participants not completing the FFQ. There was no significant difference between participants who completed and who did not complete the FFQ in terms of the prevalence of radiographic knee OA, JSN and OST, and the characteristics including age, sex and BMI.

### Assessment of dietary and non-dietary exposures

Dietary intake was evaluated using a semi-quantitative food frequency questionnaire (SFFQ) that was specially designed for the population in Hunan province in China. This SFFQ contains 63 food items that are popularly consumed in Hunan province. Participants were requested to answer how frequently (never, once per month, two to three times per month, one to three times per week, four to five times per week, once per day, twice per day, or three times and more per day) they consumed each food item during the past year. There are six options for the average amount of food consumption for each time category: less than 100g, 100–200g, 201–300g, 301–400g, 401g–500g, and more than 500g. Color pictures showing food samples with labeled weights were given to participants as a reference. The SFFQ was self-administered or completed via interview by professional researchers. We ensured the validity of using the SFFQ by comparing it with the 24-h dietary recall method, wherein tested samples were randomly selected for the same study population. The correlation coefficient between the SFFQ and the 24-h recall test for measuring Mg intake was 0.53. The Chinese Food Composition Table [34] was referenced to calculate the individual composition of macronutrients and micronutrients in the included foods.

The weight and height of each subjects were measured respectively to calculate the BMI. Participants were also asked about their average frequency of physical activity (never, one to two times per week, three to four times per week, five times and above per week) and average duration of physical activity (within half an hour, half an hour to one hour, one to two hours, more than two hours). The smoking and alcohol drinking status were asked face to face. All blood samples were drawn after a 12-hour overnight fast and were kept at 4°C until analysis. The blood fasting glucose was measured using the glucose oxidase enzyme method. Subjects with the fasting glucose ≥ 7.0 mmol/L were regarded as diabetes patients, and subjects with the systolic blood pressure ≥ 140 mm Hg or diastolic blood pressure ≥ 90 mm Hg were regarded as hypertension patients.

### Assessment of radiographic knee OA

All subjects included in this study were undergoing weight-bearing bilateral anteroposterior radiography of the knee. Two orthopedists, blinded to subjects’ clinical symptoms, assessed the radiographs independently by using the Kellgren-Lawrence (K-L) radiographic atlas. Inconsistent opinions, if any, were resolved through discussions. The severity of OA was classified into five levels according to the K-L Grade: 0 = absence of OA; 1 = suspected OA; 2 = minimal OA; 3 = moderate OA; 4 = severe joint OA [35]. A participant would be diagnosed with radiographic knee OA if at least one of his/her knee joint was graded K-L 2 or above. In addition, JSN and OST were assessed individually based on a scale of 0–3 (0 = normal; 3 = most severe) according to the Osteoarthritis Research Society International (OARSI) atlas [36].

### Statistical analysis

The quantitative data are expressed as mean ± standard deviation, and the qualitative data are expressed in percentage. The Mg intake was classified into five categories based on the quintile distribution: ≤ 218.00, 218.01–300.00, 300.01–399.00, 399.01–544.00 and ≥ 544.01 mg/day. Differences in continuous data were evaluated by the one-way classification ANOVA (normally distributed data) or the Kruskal-Wallis H test (non-normally distributed data), while differences in qualitative data were assessed by the χ^2^ test. The odds ratios (ORs) with 95% confidence intervals (CIs) for the association between radiographic knee OA, JSN, OST and dietary magnesium were calculated for each quintile of Mg intake, respectively, and the quintile with the lowest value was regarded as the reference category. In order to calculate the adjusted OR of each quintile of Mg intake, a multivariable model were adopted in the logistic analyses. Covariant variables includes age, gender, BMI, education level, smoking status, drinking alcohol status, activity level, diabetes, hypertension, energy, protein, fiber, iron, zinc, calcium intake, and nutritional supplementary. Tests for linear trends were conducted based on logistic regression using a median variable of Mg level in each category. All data analyses were performed using SPSS 17.0; a *P* value equal to or less than 0.05 was considered to be statistically significant.

## Results

The characteristics of the study population in terms of the quintiles of the total dietary Mg intake are shown in Table 1. Among this Chinese population, significant differences were observed across all quintiles of Mg intake regarding age, sex, body mass index (BMI), smoking status, alcohol drinking status, diabetes, educational level, nutrients’ supplementation, and the intake of energy, fiber, protein, zinc, iron, and calcium. No clear unadjusted association was observed between Mg intake and physical activity level, diabetes, or hypertension.

**Table 1:** Characteristics among 1626 participants according to quintiles of total magnesium intake.

	Quintile of Mg intake					
Characteristics	1 (lowest)	2	3	4	5 (highest)	*P* ^#^
Median Mg intake (mg/d)	170.3	258.5	343.9	464.1	672.2	-
Age (years)	54.0 (8.3)	52.3 (8.1)	52.3 (7.9)	52.8 (8.4)	51.9 (7.1)	0.02
BMI (kg/m^2^)	23.9 (3.2)	24.7 (3.2)	24.3 (3.2)	24.7 (3.1)	24.9 (3.0)	0.00
Female (%)	63.1	46.1	44.4	39.4	39.2	0.00
Smoking (%)	20.7	24.8	25.9	30.8	24.4	0.06
Alcohol drinking (%)	21.6	33.0	37.0	39.0	42.3	0.00
Activity level (h/d)	0.45 (0.63)	0.38 (0.57)	0.41 (0.57)	0.41 (0.57)	0.39 (0.51)	0.17
Mean total energy intake (kcal/d)	1043.0 (371.0)	1364.5 (324.6)	1635.3 (427.0)	1918.2 (549.4)	2767.2 (1125.3)	0.00
Mean fiber intake (g/d)	5.9 (3.0)	10.8 (3.7)	15.9 (4.9)	23.6 (7.4)	38.9 (22.6)	0.00
Nutrients supplementation (%)	24.0	23.9	33.6	32.7	41.0	0.00
Mean Zn intake (mg/d)	13.6 (3.5)	17.0 (2.8)	19.5 (3.6)	22.5 (4.4)	30.4 (10.0)	0.00
Mean Ca intake (mg/d)	192.2 (73.5)	338.0 (95.8)	453.9 (133.7)	632.2 (158.4)	1035.4 (572.9)	0.00
Mean Fe intake (mg/d)	15.7 (5.6)	23.0 (5.0)	30.0 (7.4)	39.0 (10.0)	59.2 (32.6)	0.00
Mean protein intake (g/d)	31.5 (9.7)	49.1 (9.9)	64.1 (13.0)	83.7 (15.1)	130.6 (56.4)	0.00
High school background or above (%)	33.9	43.6	54.0	51.1	58.0	0.00
Diabetes (%)	6.6	7.3	7.1	8.3	9.3	0.73
Hypertension (%)	24.6	30.3	27.5	29.2	26.5	0.51

Data are mean (Standard Deviation), unless otherwise indicated; Mg, magnesium; Ca, calcium; K, potassium; Zn, zinc; Fe, iron.

^#^ *P* values are for test of difference across all quintiles of magnesium intake.

The overall prevalence of radiographic knee OA among the participants in this cross-sectional study (age range 40–83 years, average 50.9±9.5 years) was 25.2%. A significant association between Mg intake and radiographic knee OA was observed in the multi-variable model, which was adjusted for age, BMI, sex, education level, smoking status, alcohol drinking status, activity level, diabetes, hypertension, total energy intake, fiber intake, iron intake, zinc intake, protein intake, calcium intake and nutrients supplementation (Table 2). The relative odds of developing radiographic knee OA were decreased by 0.53 times in the third quintile of Mg intake [odds ratio (OR) 0.52, 95% confidence interval (CI) 0.28–1.01], 0.40 times in the fourth quintile (OR 0.40, 95% CI 0.17–0.94), and 0.34 times in the fifth quintile (OR 0.34, 95% CI 0.11–1.00) compared with those in the lowest quintile, while *P* for trend was 0.111.

**Table 2:** Multivariable-adjusted relations of dietary of magnesium intake and radiographic knee OA (n = 1626).

	Quintiles of Mg intake					*P* for trend
	1 (lowest)	2	3	4	5 (highest)	
Mg intake (mg/d)	170.32	258.48	343.89	464.14	672.20	-
Participants (n)	358	358	360	360	361	-
K-L Knee OA (n)	96	91	78	81	79	-
Multi-variable adjusted ORs*	1.00 (Reference)	0.89 (0.56–1.41)	0.53 (0.28–1.01) ^##^	0.40 (0.17–0.94) ^##^	0.34 (0.11–1.00) ^##^	0.111
*P* values	-	0.608	0.052	0.036	0.049	-
Knee JSN (n)	116	99	82	104	82	-
Multi-variable adjusted ORs*	1.00 (Reference)	0.68 (0.45–1.04)	0.49 (0.28–0.88) ^##^	0.65 (0.30–1.41)	0.37 (0.14–0.98) ^##^	0.088
*P* values	-	0.073	0.016	0.28	0.045	-
Knee OST (n)	69	73	71	65	51	-
Multi-variable adjusted ORs*	1.00 (Reference)	1.39 (0.84–2.30)	1.50 (0.77–2.93)	1.44 (0.59–3.51)	1.28 (0.41–4.00)	0.720
*P* values	-	0.196	0.234	0.424	0.666	-

Data are adjusted OR (95% CI), unless otherwise indicated; Mg, magnesium; n, number; OA, osteoarthritis; JSN, joint space narrowing; OST, osteophyte.

*Multi-variable model was adjusted for age, BMI, gender, educational level, activity level, total energy intake, smoking status, alcohol drinking status, fiber intake, protein intake, zinc intake, calcium intake, iron intake, nutrients supplementation, diabetes and hypertension.

^##^p ≤ 0.05, relative to the lowest intake category.

The prevalence of JSN was 29.7% in this study. Table 2 presents the multi-variable adjusted associations between JSN and dietary Mg intake. The relative odds of finding JSN were decreased by 0.49 times in the third quintile of Mg intake (OR 0.49, 95% CI 0.28–0.88) and 0.37 times in the fifth quintile (OR 0.37, 95% CI 0.14–0.98) compared with those in the lowest quintile, while *P* for trend was 0.088.

The prevalence of OST was 20.2% in this study. The multi-variable analysis rejected any significant association between Mg intake and knee OST. The multivariable-adjusted ORs (95% CI) of OST across the five quintiles of Mg intake were 1, 1.39 (95% CI 0.84–2.30), 1.50 (95% CI 0.77–2.93), 1.44 (95% CI 0.59–3.51) and 1.28 (95% CI 0.41–4.00) respectively, while *P* for trend was 0.720.

## Discussion

This cross-sectional study observed an inverse association between dietary Mg intake and radiographic knee OA in the Chinese population, independent of major confounding factors. When JSN and OST were examined separately, dietary Mg intake was found to be associated only with JSN not with OST. As far as we know, this is the first study that confirmed an association between dietary Mg intake and knee OA in the Chinese population and perhaps even in the entire Asian population. It is also the first study showing that dietary Mg intake was associated only with knee JSN.

The findings of this study are consistent with those of some previous experiments [37–40]. For example, Lee et al. [37] supported the use of intra-articular Mg sulfate in an experimental rat OA model for attenuating the development of OA. In another study that focused on observing enhanced chondrocyte proliferation and redifferentiation, Feverabend et al. [38] suggested that Mg could be a useful tool in cartilage tissue engineering. Similarly, Egerbacher et al. [39] proved that a large amount of quinolone-induced damage could be reduced in vitro by Mg supplementation. The effect of Mg on cartilage repair was also supported in animal experiments, which demonstrated significantly decreased distal femur articular cartilage chondrocyte density caused by dietary Mg restriction [40].

Lower Mg intake was found to be associated with elevated serum CRP [11–14]. King et al. [11] suggested that children with Mg intake below the Recommended Dietary Allowances (RDA) were likely to have an increased CRP level. Similarly, another cross-sectional survey conducted on an American population confirmed that most of the participants over 17 years of age who consumed Mg at levels below the RDA were likely to have an elevated CRP level [13]. In addition, Song et al. [12,14] reported that Mg intake was inversely associated with CRP concentrations in women after adjusting a number of potentially confounding factors. Mg deficiency, causing inflammation, may have an effect on some chronic diseases [41]. Recently, a meta-analysis and systematic review covering seven cross-sectional studies (32,918 total participants) confirmed the potential benefits of Mg intake rooted in the mechanism of inflammation inhibition [10]. As is commonly known, inflammation may play an important role in the pathophysiology of OA [8,9]. Based on these analyses, the relation between low Mg intake and the high prevalence of knee OA may be explained, at least in part, by the elevated inflammation mediators.

Mg deficiency is also related to other musculoskeletal diseases [42–45]. For instance, rheumatoid arthritis patients were found to have deficient Mg intake [42–44]. A meta-analysis covering seven studies suggested that Mg deficiency was a risk factor for osteoporosis in postmenopausal women [45]. In 2012, Qin et al. [22], in a cross-sectional study, observed a modest inverse threshold association between Mg intake and radiographic knee OA in Caucasians but not in African Americans. The findings of the present study showed that such an association also exists in the Chinese population.

The present study has several strengths. First, it was adjusted for a considerable number of potentially confounding factors, especially diabetes, hypertension, and some mineral intakes, which greatly improved the reliability of the results. Second, this is the first study conducted on a large sample of the Chinese population that directly related dietary Mg intake to radiographic knee OA. Third, it is also the first study to show that dietary Mg intake is associated with knee JSN but not with the presence of OST. Finally, this study adopted the SFFQ, which is an effective method for measuring micronutrient intakes [46,47]. If a causal relation could be confirmed, it would be easy to intervene by simply changing dietary habits.

Limitations of the present study should also be acknowledged. The cross-sectional design precludes causal relations. Thus, further prospective studies and intervention trials are needed to establish a causal association between Mg intake and knee OA. As no previous research has investigated the association between Mg intake and radiographic knee OA for an Asian population, or between Mg intake and JSN or OST separately, the value of this study should not be diminished because of its cross-sectional nature. We did not perform any other Mg measurements even though the SFFQ is probably not the best questionnaire for assessing micronutrient intake. Finally, this study addressed only radiographic knee OA. Clinical OA was not investigated.

## Conclusions

This is the first epidemiological evidence that confirmed the association between Mg intake and radiographic knee OA in the Chinese population (as a segment of the Asian population), independent of some major confounding factors. When JSN and OST were examined separately, dietary Mg intake was found to be associated only with JSN, not with OST.

## Supporting Information

S1 File STROBE Statement. (PDF)
